# Chemical and Compositional Characterization of Common Wheat *Triticum aestivum* L. Blue: Implications for Innovative Food Products

**DOI:** 10.1021/acsomega.6c06063

**Published:** 2026-09-02

**Authors:** Domenico Palatucci, Michela Cariglia, Afef Ladhari, De Marco Anna, Maria Chiara Di Meo, Armando Zarrelli

**Affiliations:** † Department of Biology, 9307 University of Naples Federico II, Via Cinthia 21, 80126 Naples, Italy; ‡ Dipartimento di Economia, University of Foggia, Via Romolo Caggese 2, I-71121 Foggia, Italy; § Laboratory of Integrated Olive Production, Institut de l’Olivier, Cité Mahrajène, BP 208, 1082 Tunis, Tunisia; ∥ Department of Pharmacy, University of Naples Federico II, Via Montesano 49, 80131 Naples, Italy; ⊥ Department of Sciences and Technologies, University of Sannio, Benevento 82100, Italy; # Department of Chemical Sciences, University of Naples Federico II, Via Cintia 4, I-80126 Naples, Italy

## Abstract

There is growing interest in diversifying food sources to obtain innovative, nutrient-rich, and health-promoting products. This trend has increased scientific interest in traditional wheat varieties with improved phytochemical profiles, greater sustainability, and greater adaptability to changing environmental conditions. This study examined the chemical characteristics of *Triticum aestivum* L. Blue, a wheat variety cultivated in Southern Italy. Grains, bran, and flour were analyzed for gluten quantity and quality, ash and protein content, polyphenols, anthocyanins, proanthocyanidins, and antioxidant capacity (DPPH and ABTS). The results revealed a low gluten content (below 10%) and a relatively high protein level, potentially beneficial for pasta making. The wheat also showed more than 600 mg/100 g of total polyphenols, over 23 mg/100 g of anthocyanins, and more than 600 mg/100 g of proanthocyanidins. Its chemical composition, technological properties, and antioxidant capacity suggest that this wheat variety may be a promising ingredient for the development of innovative cereal-based products.

## Graphical abstract

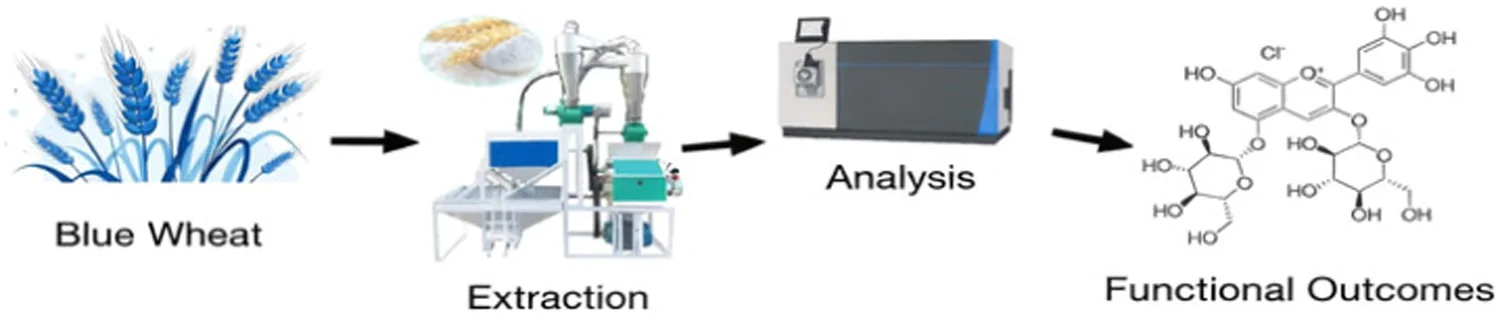

## 1 Introduction

Common wheat (*Triticum aestivum* L.), also referred to as bread wheat, is the most widely cultivated wheat species, second only to maize, and is present on all continents. In recent years, global production of common wheat, intended for human and animal consumption or industrial use, has ranged between 700 and 750 million tons, accounting for approximately 35% of the world cereal production.^1^ Although European and global agriculture have made significant advancements, the current strategy has led to dependency on mineral fertilizers, high energy consumption, reduced genetic variability, and diminished biodiversity. This increases the vulnerability of crops to biotic and abiotic stressors, with negative environmental impacts. Compared to conventional common wheat, ancient wheat varieties, sometimes referred to as minor wheats, have been shown to maintain higher concentrations of micronutrients, thrive in poor soils, and require fewer external inputs.^2–4^ Each of these underutilized cereals possesses a unique set of nutritional and bioactive compounds that industrial food enrichment cannot replicate. Unsurprisingly, organic food producers, consumers, and increasingly conventional farmers are placing high value on these crops.

The blue-grained bread wheat (*Triticum aestivum* L.) genotype analyzed in this study was supplied by Agrismarter (Foggia, Italy), which currently maintains and propagates this germplasm. The material was commercially identified by the supplier as “*T. aestivum* Blue”. Whole grain samples were provided directly by the supplier and used for the compositional and technological analyses described in this study. Local cereal varieties are characterized by hereditary traits that enhance their local adaptability and productivity in sustainable agriculture,^5^ offering a specific profile of chemical, nutritional, and physical properties. These attributes meet the increasingly specific demands of consumers while also lending themselves to industrial valorisation. Although pigmented wheat varieties have been widely investigated, information on the compositional and technological characteristics of the *Triticum aestivum* L. Blue cultivar cultivated under Southern Italian growing conditions is still limited. Furthermore, this study provides an integrated characterization of grain, flour, and bran, combining technological quality parameters with phytochemical composition and antioxidant activity. This comprehensive approach contributes to a better understanding of the distribution of bioactive compounds among milling fractions and the potential of this genotype for the development of value-added cereal-based products.

## 2 Materials and Methods

### 2.1 Materials

Seeds, bran, and flour of the common wheat (*T. aestivum* L. Blue) were supplied by the Granomischio company (Italy) in October 2023. Chemicals, analytical grade reagents, HPLC grade solvents and standards of compounds were purchased from Merck (Darmstadt, Germany).

### 2.2 Determination of Dry Gluten

Gluten was extracted from the flour (*n* = 4) following the procedure described in AACC (2005).^6^ Gluten content was expressed as dry gluten per 100 g of material.

### 2.3 Determination of Gluten Index

The gluten index, a measure of gluten quality and dough workability, was determined according to AACC (2005).^6^

### 2.4 Determination of Total Protein Content

Quantitative determination of total proteins was performed using the Kjeldahl method.^7^ Protein content was determined in quadruplicate (*n* = 4), and the results are expressed as mean ± standard deviation (SD).

### 2.5 Determination of Ash

Ash content, referring to 100 g of dry matter, was measured following the method reported by Ismai^8^ and repeated five times.

### 2.6 Extraction of Plant Matrix Using Solvents with Increasing Polarity

Approximately 20 g of sample (whole wheat, grain, flour, or bran) were finely ground using an electric grinder with the addition of dry ice. Sequential extraction was performed using 200 mL each of petroleum ether, dichloromethane, dichloromethane/methanol (1:1), and methanol for 24 h under magnetic stirring at ambient temperature and pressure in the dark. Extracts were filtered, vacuum-dried at 40 °C, and stored in a freezer.^9^ The sequential extraction with solvents of increasing polarity was adopted to recover phytochemicals with a broad range of polarities and obtain a comprehensive phytochemical profile.

### 2.7 Measurement of Total Polyphenol Content

The total polyphenol content in the extracts obtained with organic solvents was quantified using the Folin-Ciocalteu method with gallic acid as the standard.^10^ Each extract was solubilized in 5 mL of the solvent used for its extraction using a sonicator. One mL was filtered through a Phenex filter (0.45 μm) and diluted to a total volume of 15 mL. The samples (*n* = 5) and standard solutions were analyzed at 765 nm.^11^ Specifically, 0.2 mL of the sample solution (or standard or Milli-Q water for the blank) was mixed with 0.8 mL of Milli-Q water and 0.2 mL of the Folin-Ciocalteu reagent. After a 5 min incubation, 0.8 mL of Milli-Q water and 2 mL of an 8% aqueous Na_2_CO_3_ solution were added. A standard curve was constructed using gallic acid at concentrations ranging from 4.0 to 400 μg/mL, with an acceptance threshold of *r* = 0.98. Results were expressed as milligrams of gallic acid equivalents (GAE). Each extract was analyzed in quadruplicate (*n* = 4).

### 2.8 Determination of Anthocyanin Content

The total anthocyanin extract was prepared as described by Hosseinian et al.^12^ and quantification was performed spectrophotometrically,^13^ using cyanidin-3-glucoside as the standard at concentrations ranging from 1 to 200 μg/mL. Results were expressed as grams of cyanidin-3-glucoside equivalents (Cy-3-glc eq) per gram of dry material. The standard curve equation was *y* = 0.0031*x* + 0.0263, where *y* is the absorbance at 535 nm and *x* is the standard concentration. Anthocyanin determinations were conducted in quadruplicate (*n* = 4), and the results are expressed as mean ± standard deviation (SD).

### 2.9 Determination of Proanthocyanidin Content

Proanthocyanidin extraction was performed as reported by Siebenhandl et al.^13^ and the content was determined spectrophotometrically,^14^ using catechin as the standard at concentrations ranging from 0.1 to 1.0 mg/mL. Results were expressed as grams of catechin equivalents (CE) per gram of dry material. The standard curve equation was *y* =0.0028*x* + 0.0211, where *y* is the absorbance at 510 nm and *x* is the standard concentration.

### 2.10 Proanthocyanidin UHPLC Conditions

The UHPLC-HRMS setup comprised an LTQ Orbitrap XL mass spectrometer paired with an Accela 1250 binary pump, a PAL HTC Accela TMO autosampler, a PDA detector (ThermoScientific, San Jose, CA). Separation was conducted on a UHPLC column (200 × 2.1 mm^2^ i.d., 1.9 μm, Hypersil Gold AQ RP-C18) (ThermoScientific), at a flow rate of 0.3 mL/min. The mobile phase included solvent A (0.1% formic acid in water, *v*/*v*) and solvent B (0.1% formic acid in acetonitrile, *v*/*v*). The linear gradient was from 4 to 20% B (*v*/*v*) at 40 min, to 35% B at 60 min, and to 100% B at 61 min and held at 100% B to 65 min. The PDA collected spectra from 200 to 700 nm, providing real-time monitoring at 280 and 330 nm. The HRMS was operated in the negative ionization mode using the following conditions: sheath gas at 70 (arbitrary units), aux and sweep gas at 15 (arbitrary units), spray voltage at 4.8 kV, capillary temperature at 300 °C, capillary voltage at 15 V, and tube lens at 70 V. The mass range was from *m*/*z* 50 to 2000 with a resolution of 1500.^15^

### 2.11 Anthocyanin HPLC Analysis

1100 system, featuring a DAD (Agilent Technologies Inc., Palo Alto, CA), was employed for sample analysis. A Luna 5 μm C18(2) (150.0 × 4.6 mm^2^) column, along with a guard column, both supplied by Phenomenex (Torrance, CA), were utilized. Absorbance spectra were recorded for all peaks. The solvent flow rate was maintained at 1.0 mL/min, with an injection volume of 25 μL. Solvent A was 10% acetic acid and 1% phosphoric acid (*v*/*v*) in water, while solvent B contained pure acetonitrile. The equipment employed a linear gradient from 2 to 20% solvent B in 25 min; then a linear gradient of solvent B from 20 to 40% in 5 min, with simultaneous detection at wavelengths of 280 and 520 nm.^16,17^

### 2.12 Radical DPPH Scavenging Capacity

The DPPH scavenging assay is commonly used in the study of antiradical activity in vitro and for the evaluation of free radical scavenger activity. The antioxidant activity was evaluated as described with minor modifications.^18^ In particular, the radical scavenging capacity of soluble and insoluble extracts was evaluated with the stable radical 2,2-diphenyl-1-(2,4,6-trinitrophenyl)­hydrazyl (DPPH). One mL of extraction solvent with different extract dilutions was added to two mL of DPPH in methanol (5 × 10^–5^ M). The reaction mixture was incubated at 28 °C for 25 min. After 30 min, the absorbance value reached a constant value, which was used to calculate the percentage of residual DPPH. Radical reduction by antioxidants was monitored by measuring the absorbance at 517 nm using a JASCO V-750 UV-Visible Spectrophotometer (Jasco Europe, Milan, Italy). Five extracts were analyzed for each sample, each with four different dilutions. A dose–response line was calculated for each sample, and the amount of whole meal flour needed to eliminate 50% of the radical was determined (IC_50_). A regression line was also calculated for the reference antioxidant Trolox, with concentrations ranging from 3 to 50 μM. The antioxidant activity was expressed as the ratio between the IC_50_ of Trolox and the IC_50_ of the sample, that is, millimoles of Trolox equivalent (TE) per kilogram of dry weight.

### 2.13 Radical ABTS Scavenging Capacity

This test determines the antioxidant capacity of various biological matrices (aqueous extracts of foods, beverages, fruit/vegetable juices, plasma, saliva, etc.) through the reaction between the sample under analysis and a cationic radical. In this assay, the colored and stable cationic radical (ABTS^+•^) is generated via the oxidation of the diammonium salt of 2,2′-azino-bis­(3-ethylbenzothiazoline-6-sulfonic acid) (ABTS) using a potassium persulfate solution, K_2_S_2_O_8_.^19^ The cationic radical shows an absorption peak at 734 nm, which decreases as antioxidant compounds donate a hydrogen atom or an electron, after a predetermined incubation time. This decrease (decolorization) is proportional to the antioxidant charge present in the sample. The quantification of the antioxidant activity of a given compound/extract is derived from a calibration curve correlating the absorbance decrease with the concentration of the antioxidant. Antioxidant activity is expressed in Trolox equivalents (TE). A stock solution of non-radical ABTS was prepared at a concentration of 10^–4^ M in 50 mM acetate buffer (pH 4.5). All kinetics were conducted in triplicate by reacting 2 mL of the 10^–4^ M ABTS^+•^ solution with 50 μL of the sample solution in a quartz cuvette for approximately 20 min.

### 2.14 Statistical Analysis

Data are reported as means and standard deviation (SD). The normality of the data distribution was assessed by the Shapiro-Wilk test. One-way analysis of variance (ANOVA) was employed for mean separation considering as threshold of statistical significance a *P*-value lower than 0.05. ANOVA was performed to evaluate statistically significant differences between bran, flour and grain. Calculations were performed with the SigmaPlot 12.2 software (Systat Software, San Jose, CA).

## 3 Results

### 3.1 Dry Gluten, Gluten Index, Protein and Ash Content

The analyzed wheat variety exhibited a dry gluten percentage of 9.7 ± 0.10 and a gluten index of 57 ± 2.1 (Table 1). The gluten index value falls within the range generally associated with satisfactory dough strength and processing performance, indicating suitable technological properties of the flour. However, this parameter reflects gluten quality rather than its physiological effects or tolerability. It should be noted that wheat varieties with higher gluten index values are typically preferred for pasta production.^20^ The protein content is 16.4% dry weight, exceeding the 14% threshold required to classify the corresponding flour as special. The protein content observed in the present study is comparable to, or slightly higher than, values reported for other pigmented bread wheat cultivars, supporting the favorable technological characteristics of this genotype.^21^

**Table 1:** Dry Gluten (%), Gluten Index, Protein (% Dry Weight, DW), and Ash Content (% DW) of Flour from *T. aestivum* Blue^a^

dry gluten	gluten index	protein	ash content
9.7 ± 0.10	57 ± 2.1	16.4 ± 1.2	1.19 ± 0.11

a Data are expressed as mean ± SD (*n* = 4).

In general, protein content is considered more important than gluten strength, as it is more closely related to the positive characteristics of dried pasta, although this relationship is influenced by processing methods and gluten composition.^22^

The ash content was 1.19%, which is relatively low compared to other varieties available on the market. This parameter, generally linked to genetic and environmental factors and influenced by protein content,^23^ is also considered a quality attribute.^24^

### 3.2 Polyphenol Content, Anthocyanins, and Proanthocyanidins

Polyphenols are a large group of chemically diverse secondary metabolites known for their antioxidant properties and their potential contribution to the nutritional quality of cereal products.^25,26^ They partly accumulate in the outer layer of the grain, serving a predominantly non-nutritive function.^27^ The most well-known and abundant include derivatives of cinnamic acid, flavonoids, and lignans.

The finely powdered material (whole wheat) was extracted using various solvents, with the relative percentage yields of the obtained extracts summarized in Table 2. The highest extraction yields were obtained with petroleum ether and, particularly, with a 1:1 mixture of methylene chloride and methanol.

**Table 2:** Extraction Yield (%) and Total Phenolic Content (mg GAE/g Extract) of Sequential Extracts Obtained from *T. aestivum* Blue^a^

sample	petroleum ether	CH_2_Cl_2_	CH_2_Cl_2_/CH_3_OH (1:1)	CH_3_OH
extraction yield %	1.5 ± 0.06b	0.9 ± 0.02c	2.9 ± 0.08a	0.9 ± 0.03c
total phenolic content (mg GAE/g extract)	1.7 ± 0.03c	14.5 ± 0.16a	14.1 ± 0.20a	12.7 ± 0.21b

a Data are expressed as mean ± SD (*n* = 4). Significant differences among extraction indicated by lowercase letters (*ANOVA*, *p* < 0.05).

Each extract was analyzed in triplicate using the Folin–Ciocalteu reagent. Total phenolic content was expressed as GAE per 100 g dry weight (DW), and the reported values represent the mean ± standard deviation of four independent measurements (Table 2). The total phenolic content was lowest in the least polar extract (petroleum ether), whereas similar values were observed among the three more polar extracts.

The distribution of total polyphenols, anthocyanins, and proanthocyanidins among grain, flour, and bran is reported in Table 3. Overall, bran showed the highest concentrations of all three classes of bioactive compounds, followed by whole grain, whereas flour contained the lowest levels.

**Table 3:** Total Polyphenols (μg GAE/g DW), Anthocyanins (μg Cy-3-glc/g DW), and Proanthocyanidins (μg CE/g DW) in Grain, Flour, and Bran of *T. aestivum* Blue^a^

sample	grain	flour	bran
total polyphenols (μg GAE/g DW)	2229 ± 66b	934 ± 14c	3018 ± 338a
anthocyanins (μg Cy-3-glc eq/g DW)	103.0 ± 12.3b	47.3 ± 6.3c	181.6 ± 3.9a
proanthocyanidins (μg CE/g DW)	1852 ± 100b	421 ± 89c	4546 ± 665a

a AData are expressed as mean ± SD (*n* = 4). Significant differences between bran, flour, and grain are indicated by lowercase letters (*ANOVA*, p < 0.05).

More noteworthy are the data regarding total polyphenol content, expressed as micrograms of gallic acid per gram of dry sample, in wheat grain, flour, and bran of *T. aestivum*, analyzed separately (Table 3). The highest content was clearly found in the bran, with 35% more than in the grain and 220% more than in the flour.

A similar trend was observed for the total content of anthocyanins and proanthocyanidins, expressed as micrograms per gram of dry sample. Bran contained almost 80% more anthocyanins than the grain and nearly three times as much (285%) as the flour.

The difference was even greater regarding proanthocyanidin content, with bran containing 2.5 times more than the grain and nearly 11 times more than the flour.

The total phenolic content was consistent with the range reported for pigmented wheat varieties and agrees with previous studies showing higher phenolic levels in pigmented wheat than in conventional white wheat.^13,21^

### 3.3 Chemical Composition

The HPLC analysis determined the content of the main anthocyanins (glucosylated compounds) in wheat grain, flour, and bran of *T. aestivum* (Table 4). The most abundant compound was Delphinidin 3-rutinoside, while Delphinidin 3-glucoside and Petunidin 3-glucoside were present in amounts less than 50% of the former but approximately double those of Cyanidin 3-glucoside and Peonidin 3-glucoside, regardless of the plant matrix considered. Even lower quantities of Malvidin 3-glucoside were detected. The anthocyanin content observed in the present study is consistent with values reported for other blue wheat cultivars. As previously described, these pigments are predominantly localized in the aleurone layer, explaining their higher concentration in bran fractions.^21,28^

**Table 4:** HPLC Determination of Anthocyanidin Glycosides (μg/g DW) in Grain, Flour, and Bran of *T. aestivum* Blue^a^

anthocyanins	grain	flour	bran
delphinidin 3-glucoside	15.50 ± 0.10b	6.90 ± 0.30c	30.30 ± 2.18a
delphinidin 3-rutinoside	36.50 ± 0.30b	15.30 ± 0.50c	72.51 ± 0.71a
cyanidin 3-glucoside	6.10 ± 0.10b	1.25 ± 0.01c	11.56 ± 0.84a
petunidin 3-glucoside	13.75 ± 0.05b	4.05 ± 1.35c	25.19 ± 2.09a
peonidin 3-glucoside	8.45 ± 0.05b	1.78 ± 0.48c	12.30 ± 0.80a
malvidin 3-glucoside	1.01 ± 0.10b	2.10 ± 0.40b	7.29 ± 0.19a

a Data are expressed as mean ± SD (*n* = 4). Significant differences between bran, flour, and grain are indicated by lowercase letters (*ANOVA*, *p* < 0.05).

The analysis of anthocyanidins (aglyconic compounds) revealed the almost exclusive presence of Delphinidin in flour, whereas Malvidin was the predominant compound in grain and bran, with smaller quantities of Delphinidin, Cyanidin, Petunidin, and Peonidin detected (Table 5).

**Table 5:** HPLC Determination of Anthocyanidins (μg/g DW) in Grain, Flour, and Bran of *T. aestivum* Blue^a^

anthocyanidins	grain	flour	bran
delphinidin	0.77 ± 0.07b	31.38 ± 1.32a	1.41 ± 0.40b
cyanidin	0.49 ± 0.07a	-	0.69 ± 0.01a
petunidin	1.33 ± 0.07a	-	1.20 ± 0.10a
peonidin	0.39 ± 0.03b	-	1.63 ± 0.07a
malvidin	84.29 ± 0.45b	-	164.08 ± 1.20a

a Data are expressed as mean ± SD (*n* = 4). Significant differences between bran, flour, and grain are indicated by lowercase letters (*ANOVA*, *p* < 0.05).

### 3.4 Antioxidant Activity

To provide a more comprehensive evaluation of antioxidant capacity, two complementary assays, DPPH and ABTS, were performed (Table 6). Results were expressed as μmol Trolox equivalents (TE) per gram of dry weight. In the DPPH assay, bran showed the highest antioxidant capacity, with values approximately 20% higher than those of grain and nearly 50% higher than those of flour. Similarly, the ABTS assay revealed the highest antioxidant activity in bran, with values approximately fourfold higher than those observed for grain and flour. The higher antioxidant capacity of bran is consistent with its higher phenolic and anthocyanin contents and agrees with previous studies reporting a greater antioxidant potential of pigmented wheat than conventional wheat.^13,21^

**Table 6:** Antioxidant Capacity Determined by DPPH and ABTS Assays (μmol TE/g DW) in Grain, Flour, and Bran of *T. aestivum* Blue^a^

method	grain	flour	bran
DPPH (μmol TE/g DW)	9.64 ± 0.39b	8.35 ± 0.10b	12.12 ± 0.17a
ABTS (μmol TE/g DW)	1.70 ± 0.63b	2.13 ± 0.09b	7.42 ± 0.08a

a Data are expressed as mean ± SD (*n* = 4). Significant differences between bran, flour, and grain are indicated by lowercase letters (*ANOVA*, *p* < 0.05).

Overall, the results indicate that *Triticum aestivum* L. Blue exhibits a chemical and technological profile of particular interest for the development of innovative food products.

Its relatively low gluten content, together with a gluten index indicative of satisfactory dough workability, describes the technological characteristics of this flour. However, these parameters do not allow conclusions to be drawn regarding nutritional or physiological effects, and further studies are required to evaluate any potential clinical relevance of its gluten profile. At the same time, the high protein content exceeds the levels typically required for high-quality productions and may positively influence the technological properties of pasta. The abundant presence of phenolic compounds, anthocyanins, and proanthocyanidins, combined with the strong antioxidant activity observed across all analyzed matrices, supports its potential as a source of bioactive compounds for cereal-based products.

This richness in secondary metabolites, together with cultivation practices based on low-input systems and slow, low-temperature processing methods, may offer considerable potential for developing foods with an enhanced phytochemical composition. Overall, *T. aestivum* L. Blue represents a promising germplasm for the development of value-added cereal products. This study provides a comprehensive characterization of this blue wheat genotype and contributes additional information on its technological and phytochemical features under Southern Italian growing conditions. However, the findings refer to a single genotype cultivated under specific environmental conditions. Therefore, further studies involving multiple genotypes, growing environments, and growing seasons are required to assess the relative contributions of genetic and environmental factors to the observed compositional and technological characteristics.
